# Blocking Protein Phosphatase 1 [PP1] Prevents Loss of Tether Elasticity in Anaphase Crane-Fly Spermatocytes

**DOI:** 10.3389/fmolb.2021.636746

**Published:** 2021-06-08

**Authors:** Arthur Forer, Aisha Adil, Michael W. Berns

**Affiliations:** ^1^Biology Department, York University, Toronto, ON, Canada; ^2^Beckman Laser Institute, University of California, Irvine, Irvine, CA, United States; ^3^Department of Biomedical Engineering, University of California, Irvine, Irvine, CA, United States; ^4^Department of Surgery, University of California, Irvine, Irvine, CA, United States; ^5^Department of Developmental and Cell Biology, University of California, Irvine, Irvine, CA, United States; ^6^Department of Bioengineering, University of California, San Diego, San Diego, CA, United States; ^7^Institute of Engineering in Medicine, University of California, San Diego, San Diego, CA, United States

**Keywords:** mitotic tethers, calyculin A, PP1, crane fly spermatocytes, anaphase movements, laser irradiations

## Abstract

In normal anaphase cells, telomeres of each separating chromosome pair are connected to each other by tethers. Tethers are elastic at the start of anaphase: arm fragments cut from anaphase chromosomes in early anaphase move across the equator to the oppositely-moving chromosome, telomere moving toward telomere. Tethers become inelastic later in anaphase as the tethers become longer: arm fragments no longer move to their partners. When early anaphase cells are treated with Calyculin A (CalA), an inhibitor of protein phosphatases 1 (PP1) and 2A (PP2A), at the end of anaphase chromosomes move backward from the poles, with telomeres moving toward partner telomeres. Experiments described herein show that in cells treated with CalA, backwards movements are stopped in a variety of ways, by cutting the tethers of backwards moving chromosomes, by severing arms of backwards moving chromosomes, by severing arms before the chromosomes reach the poles, and by cutting the telomere toward which a chromosome is moving backwards. Measurements of arm-fragment velocities show that CalA prevents tethers from becoming inelastic as they lengthen. Since treatment with CalA causes tethers to remain elastic throughout anaphase and since inhibitors of PP2A do not cause the backwards movements, PP1 activity during anaphase causes the tethers to become inelastic.

## Introduction

Tethers are elastic connections between telomeres of separating anaphase chromosomes. They produce tension between the separating chromosome arms, stretching them by ~10% (Forer et al., 2017). Though tethers have not been identified in images of living cells, their presence was identified operationally: when an arm of an anaphase chromosome is severed, the resulting arm fragment moves rapidly across the spindle equator, led by the telomere, until it reaches the partner telomere. Tethers were originally described (operationally) in crane-fly spermatocytes (and named as such) by LaFountain et al. (2002). Their presence has also been determined in other animal cells (Forer et al., 2017). From the range of cells in which tethers are present, tethers seem to be universal force-producing components of mitotic and meiotic spindles in animal cells. In fixed and stained cells, non-DNA connections between the telomeres of separating anaphase chromosomes have been described/illustrated in large numbers of animal cell types (Paliulis and Forer, 2018), presumably representing tethers. Tethers are probably present in plant cell spindles, too, since stained connections between separating telomeres are also seen in plant cells (Paliulis and Forer, 2018), e.g., in cells from *Oenothera* (Figure 19 in Cleland, 1926) and *Haemanthus* (Figures 8C,D in Bajer and Molè-Bajer, 1986). A range of evidence (discussed, for example, in Forer et al., 2017; Forer and Berns, 2020) shows that the movements of arm fragments are not microtubule-based and require direct physical connections between separating telomeres.

Tethers connect all anaphase chromosome partners but do not connect all chromosome arms (Forer et al., 2017). In crane-fly spermatocytes in particular, only two of each chromosome's four arms are connected by tethers (LaFountain et al., 2002; Sheykhani et al., 2017). Tether elasticity decreases as tethers elongate (i.e., as the distance between separating telomeres increases); fewer and fewer arm fragments reach their partners or even reach the opposite half-spindle at longer lengths. Arm fragments with longer tethers that *do* move, move more slowly than those with shorter tethers (LaFountain et al., 2002). When tethers are severed the connected arms become shorter by about 10% of the stretched length, a constant shrinkage throughout anaphase, even at tether lengths at which arm fragments do not move (Forer et al., 2017). Thus, tethers connect all chromosomes, though not all arms; they are elastic in early anaphase, they become less elastic as tether lengths increase, and in late anaphase they are inelastic but still connect partner chromosomes.

Dephosphorylation by protein phosphatase 1 (PP1) may cause loss of tether elasticity. After anaphase cells were treated with calyculin A (CalA), single or multiple half-bivalents moved backwards after they reached the poles; the movements were led by telomeres that moved to the telomeres of their partners at the other pole, as if they were pulled by tethers (Fabian et al., 2007a). CalA inhibits protein phosphatases 1 and 2A (PP1 and PP2A) and it may be that phosphorylated tethers are elastic and dephosphorylated tethers are not (Paliulis and Forer, 2018). Kite and Forer (2020) tested this hypothesis by adding CalA to cells at different tether lengths. If blocking phosphatase activity preserves the elasticity of tethers, blocking phosphatases later and later in anaphase should result in fewer and fewer chromosomes moving backwards, because loss of tether elasticity is gradual. Kite and Forer (2020) found exactly that: whereas 80% of the chromosomes moved backwards when CalA was added at tether lengths up to 3 μm, fewer moved backwards at longer tether lengths (Table 1). They also showed that this effect is due to PP1. They showed experimentally that PP2A inhibitors did not cause backwards movements. They eliminated effects on other phosphatases by showing that the CalA concentrations used would not affect the activities of other protein phosphatases. Therefore, the effects of CalA in causing backwards chromosome movements were due solely to inhibiting PP1. Kite and Forer (2020) assumed that the backwards chromosome movements are due to tethers because the movements are led by telomeres and the telomeres move to their partner telomeres. They also assumed that phosphorylation controls tether elasticity because the phosphorylation is of the tethers themselves. However, their evidence is circumstantial, and the movements might possibly be propelled by some other interzone component(s). In this article we directly test the role of tethers by adding CalA to anaphase crane-fly spermatocytes and then either severing tethers, severing chromosome arms, or ablating telomeres.

**Table 1:** Whether chromosomes move backwards at the end of anaphase depends on when Calyculin-A is added during anaphase.

**Tether length when Percentage of chromosomes**	**CalA was added that moved backwards**
1–3 μm	80%
9–11 μm	10%
>11 μm	0%

*Data were taken from Figure 7 of Kite and Forer (2020)*.

## Methods

Crane flies (*Nephrotoma suturalis* Loew) were reared in the laboratory using methods similar to those described in Forer (1982). Testes were removed from fourth-instar larvae using methods described in Forer and Pickett-Heaps (2005) and Kite and Forer (2020). In brief, larvae were covered with halocarbon oil to prevent evaporation during the dissection. The testes were removed under oil, most of the fat was removed, the oil was rinsed off, and each testis was placed on a coverslip in a small drop of insect Ringers solution which contained fibrinogen. The testis was pierced, the cells were spread out in the fibrinogen solution, thrombin was added to form a clot to hold the cells in place, and the clot-embedded cells were placed in a perfusion chamber and rinsed with Ringers solution. We followed control cells kept in Ringers solution as well as cells treated with CalA. Laser irradiations were of tethers, chromosome arms, or telomeres, using methods described in detail in Sheykhani et al. (2017) and Forer et al. (2013). Cells treated with CalA generally were followed from metaphase. CalA was perfused into the chamber at various times after the start of anaphase. The CalA (from LC Laboratories) was dissolved in DMSO as a 100 μM stock solution and stored frozen. Before the perfusion, thawed CalA was diluted with insect Ringers solution to final concentrations between 30 and 100 nM. (The highest concentration of DMSO found in the diluted CalA, 0.1% DMSO, has no effect on chromosome movements.) Cells were followed using phase-contrast microscopy (63X, NA 1.4, Zeiss Plan Apochromatic lens). Using freeware *Irfanview*, digital images obtained throughout the experiments (recorded every 2–4 s) were trimmed, time stamped using the digital information in each file, and converted into bmp images. The images were compiled into movies using freeware *VirtualDub2*, distances were measured using an in-house program (Wong and Forer, 2003), and movement graphs were obtained using the program *SlideWrite*, as described in detail in Ferraro-Gideon et al. (2013) and Forer and Berns (2020). Portions of the cell were irradiated using a femtosecond laser in a microbeam apparatus described in Shi et al. (2012); Harsono et al. (2013), and Berns (2020). The dosimetry for laser effects was similar to that used previously (Forer et al., 2013, 2017; Harsono et al., 2013; Sheykhani et al., 2017; Forer and Berns, 2020). In our experiments the laser was tuned to either 740 or 780 nm. The laser was aimed to cut along a user-specified line in the image. Images were viewed on the computer screen, lines were drawn on the parts of image that were to be cut, and when the shutter was opened the laser cut along the lines, either in the one image plane specified, or in three planes: the image plane plus one above and one below. The three Z-planes were separated along the Z-axis by 0.5 μm. The montages in this article were compiled from individual images using Photoshop.

## Results

### General Description of CalA Effects

In control crane-fly spermatocytes, at anaphase the three autosomal bivalents disjoin and move to the pole but the two unpaired sex-chromosome univalents remain at the equator. The spindle stays at a constant length during autosomal anaphase. After the autosomes reach the poles, the sex-chromosome univalents segregate to the two poles as the spindle elongates (Forer, 1980; Kite and Forer, 2020).

CalA was added to anaphase crane-fly spermatocytes at final concentrations ranging, in different cells, from 30 to 100 nM. We used these concentrations based on the concentrations that induced backwards movements, reported by Fabian et al. (2007a). We did not see any differences in effects at the different concentrations. In most cells we added the CalA within a few minutes after seeing clear separation between all arms at the start of anaphase. In 25/29 cells the CalA caused chromosomes to move backwards after moving to the poles (**Figure 12A**). In most of these cells backward movements did not start until some minutes after the chromosomes reached the poles, but in 7 of the 25 cells the backwards movements began when chromosomes were halfway or two-thirds of the way to the poles. Sometimes individual chromosomes moved backwards, with telomere moving toward telomere, and sometimes the chromosomes at the poles fused into one or two masses that moved backwards together, led by the telomere-ends of arms that protruded from the mass and that pointed to the partner telomere at the other pole. The chromosomes at the poles often moved quickly to and from the pole before and/or as they moved backwards, as described by Fabian et al. (2007a) and Kite and Forer (2020).

Cal-A also caused other chromosome movement changes. CalA altered sex-chromosome behavior. In control cells the sex chromosomes do not move from the spindle equator until the autosomes reach the poles (e.g., Forer, 1980). In cells treated with CalA, on the other hand, both those in which the autosomes moved backwards and those in which they did not, both sex chromosomes moved erratically up and back along the spindle axis starting in mid-anaphase, continuing throughout anaphase (Fabian et al., 2007a; Kite and Forer, 2020). CalA also caused autosome poleward movements to speed up (Fabian et al., 2007a; Sheykhani et al., 2013).

### Cutting Tethers and Cutting Chromosomes After CalA Treatment

#### Cutting Tethers Stops Backwards Movements

**Figure 1:**
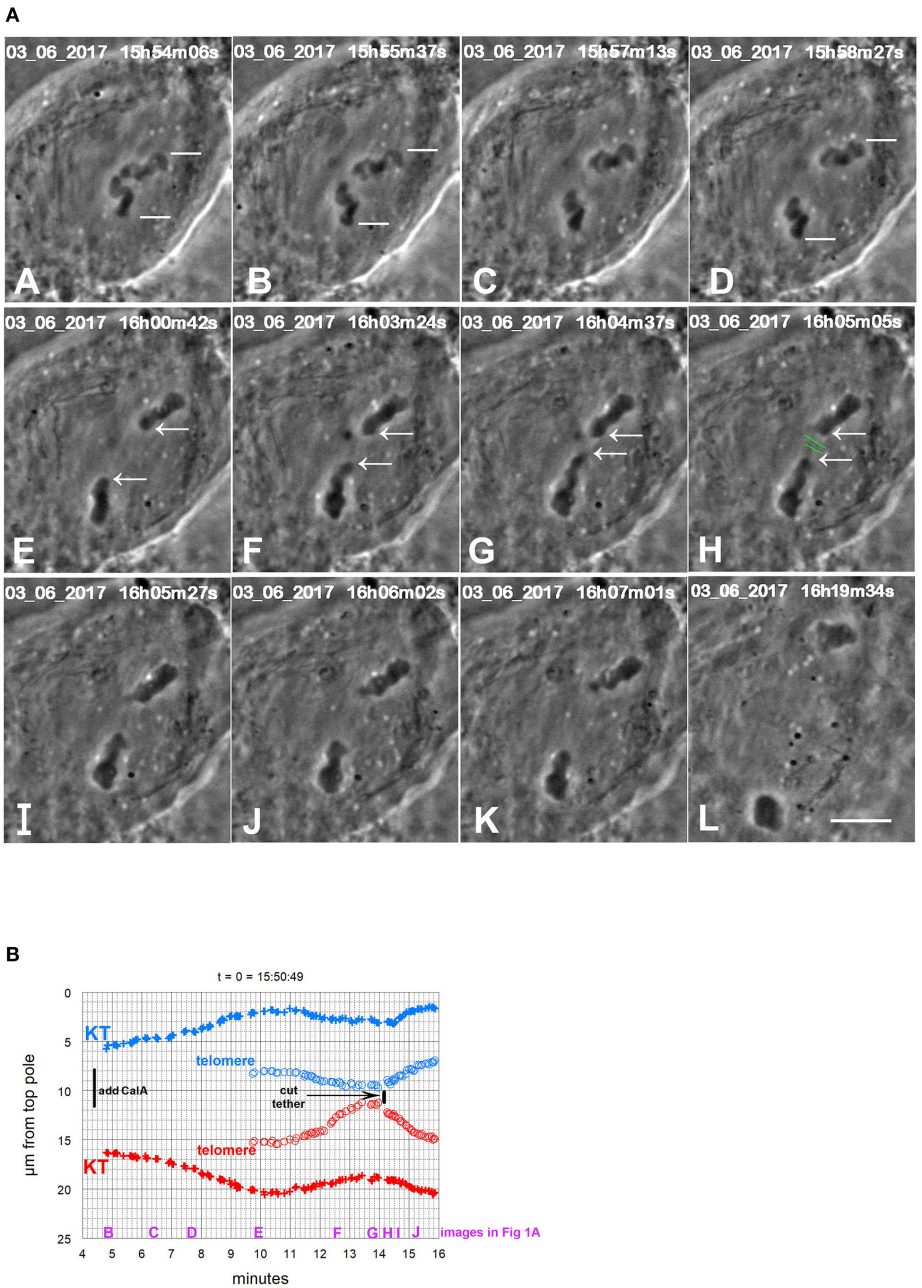
CalA-treated cell. **(A)** is a montage showing that cutting tethers stops the backwards movements. As in all other montages, the time is indicated in hours, minutes, and seconds in the upper right of each panel. Calyculin was added to the cell between (A,B). The white lines in (A–D) point to two kinetochores that lead the movements toward the poles. Poleward movements slow down in frames (D,E). The chromosomes seemed to fuse together in (E). The opposite telomeres that are closest together are indicated by arrows in (E–H). Telomeres begin to move toward each other between (E,F), and by (G) they are very close. The tethers were severed between frames (G,H) at the positions indicated by the green lines. After the tethers were severed, the chromosomes reversed direction again and moved to their original poles (H through L). The scale bar in (L) represents 5 μm in the cell. **(B)** is a graph of distance vs. time for chromosomes in the cell illustrated in **(A)**. The leading kinetochores (indicated in **A**) are labeled as (+) and the corresponding telomeres as (o). Those chromosomes moving to the bottom pole are labeled in red and those moving to the top pole in blue. Kinetochores (in this and in all other Figures) are labeled KT. To facilitate comparison with the images in **(A)**, we have put on the heading of the graph the time on the images that would correspond to *t* = 0 on the graph, and we have labeled along the abscissa the times of the images (B–J).

To test whether tethers are responsible for backwards movements of chromosomes at the end of anaphase in CalA-treated cells, we cut the interzone (between separating chromosomes) in eight CalA-treated cells as chromosomes moved backwards. The backwards movements stopped in all eight cells. In two cells the chromosomes remained stopped. In the other six cells the chromosomes reversed direction and moved toward their original poles (Figure 12B). This occurred even in cells in which tethers were cut when the partner telomeres were within a few micrometers of each other (Figure 1). Removing the backwards forces allowed the chromosomes to move forward again, indicating that poleward forces were still acting on the backwards moving chromosomes. We know from previous experiments that laser cuts in the interzone sever tethers (Sheykhani et al., 2017; Forer et al., 2018) so these experimental results support the interpretation that elastic tethers pull the chromosomes backwards. However, there may be other components in the interzone that the laser might have damaged. Since we cannot be sure that it was the cutting of tethers *per se* that stopped the backwards movements, we tested this interpretation further by cutting chromosomes directly.

#### Cutting Chromosome Arms of Backward Moving Chromosomes Stops the Backwards Movements

**Figure 2:**
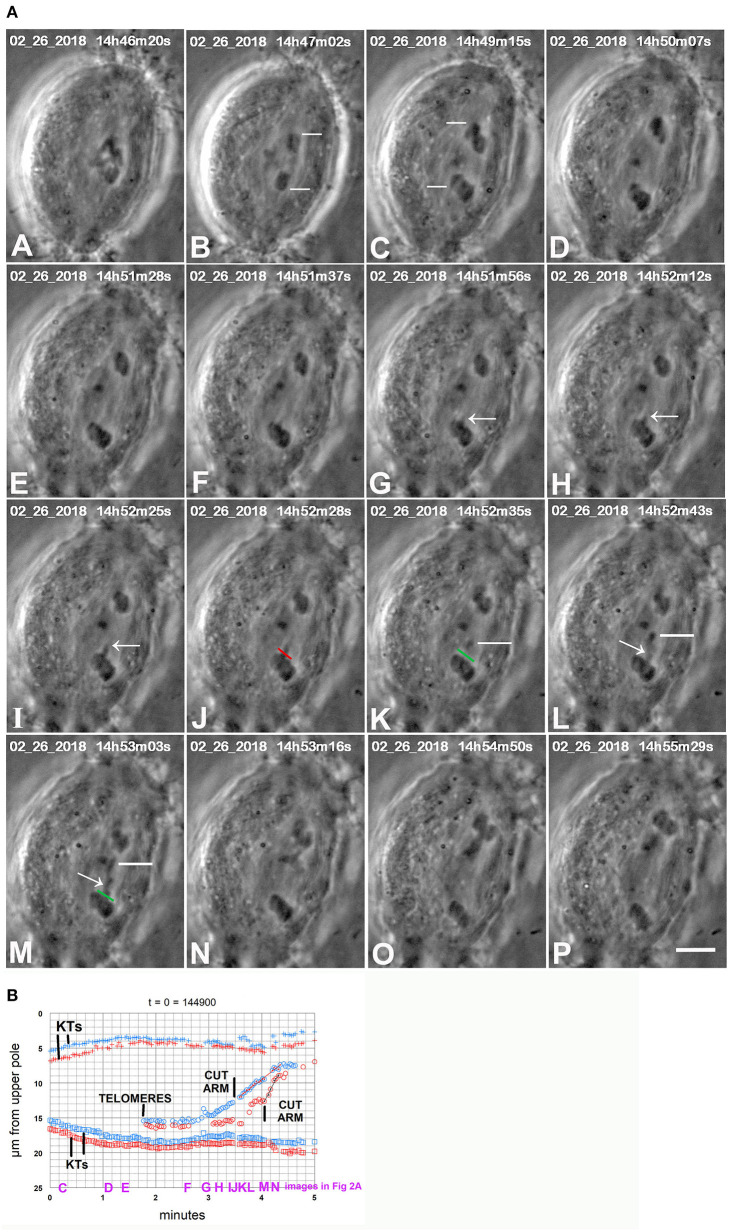
CaA-treated cell. **(A)** is a montage showing that cutting arms from backwards moving chromosomes stops the backwards movements. CalA was added to the cell between frames (A,B). Kinetochores of two separating pairs are indicated by white lines in (B,C), one pair in (B) and another in (C). Chromosomes stopped moving polewards near frame (D), and started moving backwards by frame (E). As they moved backwards one arm was pulled out from a chromosome (arrows in G,H,I). The arm was cut at the position indicated by the red line in (J) (which is at the start of the cut) and the green line in (K) (after the cut). The resultant arm fragment, indicated by white lines in (K–M), moves to the partner telomere. A second arm was pulled out (arrows in L and M) and was cut (after frame M) at the position marked in green in (M); the resultant arm fragment moved to the partner telomere. The severed arms were not visible between (M,N) because they were not in focus in the captured images. After the arms were cut the backwards movements stopped and the chromosomes began moving to the original poles. The scale bar in (P) represents 5 μm in the cell. **(B)** is a graph of distance vs. time for chromosomes in the cell illustrated in **(A)**. The two kinetochore pairs (KTs) are those indicated by white lines in (B,C) of **(A)**, one pair in red, the other in blue. The two arm cuts correspond to the ones illustrated in **(A)**. The kinetochores stop moving poleward at about 1.5 min on the graph, begin to move backwards at about 2 min, and after the arms were cut they started moving to their original poles again. To facilitate comparison with the images in **(A)**, a heading of the graph gives the time on the images that corresponds to *t* = 0 on the graph, and we have labeled along the abscissa the times of images (C–N).

Cutting arms from chromosomes removes the mechanical connection between separating partners, thereby disabling the tethers without cutting the interzone (Forer and Berns, 2020). Cutting arms when chromosomes move backward eliminates the possibility that backwards movements were stopped because of collateral damage to something other than tethers. In nine cells treated with CalA we severed arms that appeared to be leading the backwards chromosome movements. In all nine cells the backwards movements stopped (Figure 12C). In six cells the chromosomes stopped moving backwards and then reversed direction and moved in their original direction to the poles (Figure 2). In three cells the chromosomes stopped moving, and no further motion ensued. In one cell in meiosis-II, backwards moving chromosomes reversed direction and moved to the pole after an arm was cut. In all cases, the arm fragment that was formed from severing the arm moved rapidly to the partner telomere (Figure 12C). Since disabling tethers in this way stops the backwards movements, this experiment shows that the forces that pull the chromosomes backwards arise from tethers. Severing arms earlier in anaphase confirms this conclusion.

#### Cutting Chromosome Arms Before Chromosomes Move Backwards Prevents Subsequent Backwards Movements

We severed arms of chromosomes in 17 CalA-treated cells, in mid- to late-anaphase, prior to the chromosomes moving backwards. In none of the cells did the chromosomes move backwards after they reached the pole (Figure 12D). The arm fragments moved backwards to the telomeres of their partners but there was no backwards movement of chromosomes. This is quite different from the high frequency of backwards movements in cells without severed arms (Table 2). Disabling tethers in this way blocks subsequent backwards movements, which is strong evidence that tethers pull the chromosomes backwards. This in turn indicates that CalA prevents tethers from becoming inelastic as they elongate.

**Table 2:** Backwards movements of chromosomes after they reach the poles in Calyculin-A treated cells.

**Backwards chromosome movements in**			
**Cells without severed arms**		**Cells with ≥ 1 severed arm**	
Moved backwards	NONE move backwards	Moved backwards	NONE move backwards
25	4	0	17

*Data are from experiments in the current article*.

#### Ablating Telomeres

**Figure 3:**
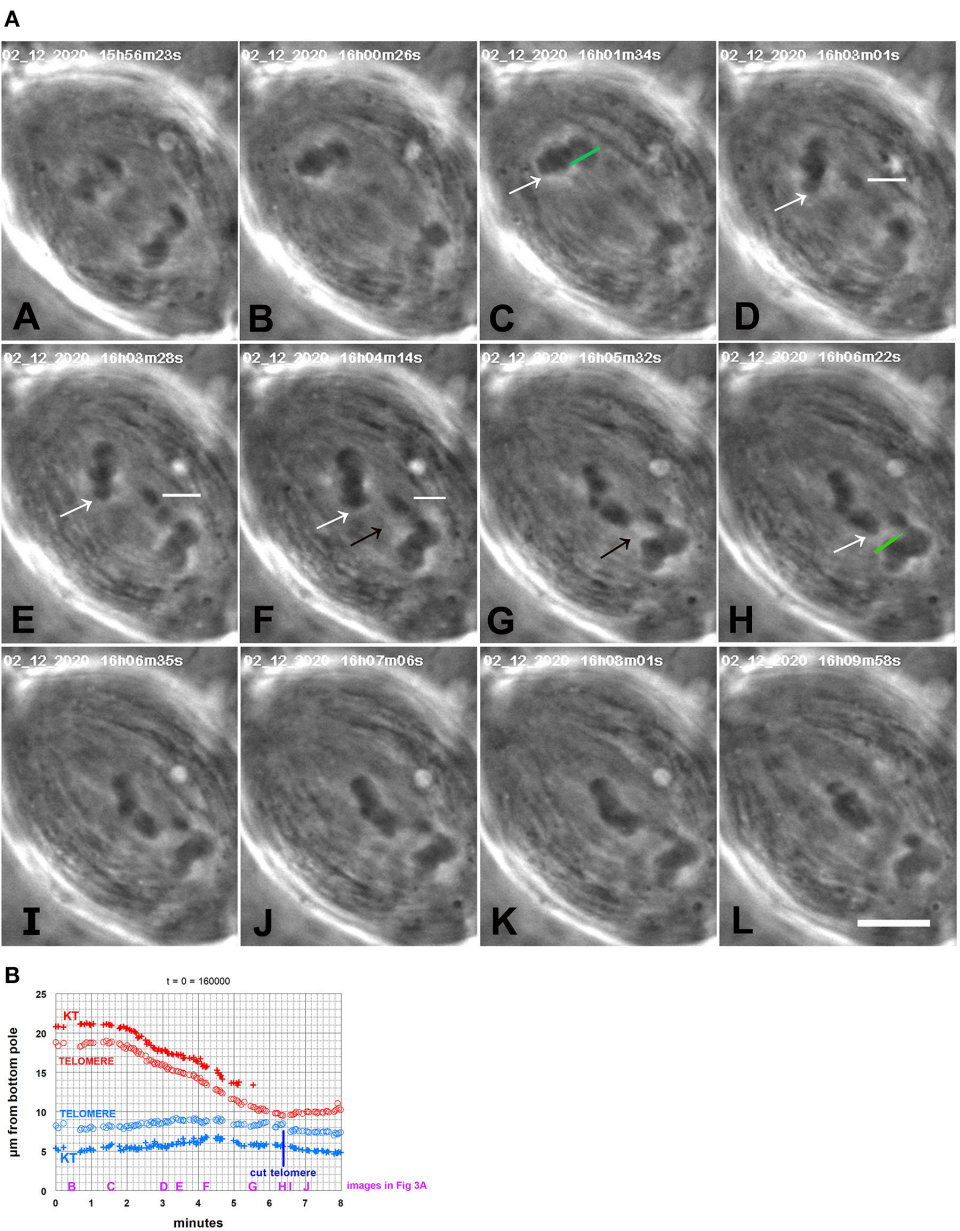
CalA-treated cell. **(A)** is a montage showing that backwards movements of chromosomes stop after cutting the telomeres to which the chromosomes are moving. CalA was added to the cell ~4.5 min before the time shown in (A). In (A,B), the half-bivalents move poleward. As they started to move backwards (C), an arm was cut (green line in C); the resultant arm fragment (indicated by a horizontal white line in D–F) moved to its partner. As the arm fragment started to move (D), a chromosome in the other group (indicated by white arrows in C–H) moved backwards toward its partner, seeming to pull attached chromosomes with it. When that chromosome neared its partner, the partner's telomere was ablated (H, green line, just before the irradiation). The backwards-moving chromosome stopped moving after the telomere was ablated (I–L). The black arrows in (F,G) point to a faint black line in each panel that extends between the telomere of the backwards-moving chromosome and the telomere of the partner; this might be an image of a tether extending between them. The scale bar in (L) represents 5 μm in the cell. **(B)** is a graph of distance vs. time of the chromosomes illustrated in **(A)**. The top chromosomes are red, the bottom blue. The measurements were of kinetochores and telomeres of the chromosome pointed to by the white arrows in **(A)** and its partner chromosome near the bottom pole, that to which the arrowed chromosome moved. After the telomere of the bottom chromosome was ablated the top chromosome stopped moving backwards and the bottom chromosome started moving toward the bottom pole. To facilitate comparison with the images in **(A)**, a heading of the graph gives the time on the images that would be equivalent to *t* = 0 on the graph, and we have labeled along the abscissa the times of images (B–J).

Part of the original argument showing that tethers are attached to telomeres derives from experiments in otherwise not-treated cells that showed that arm fragments stopped moving after laser ablation of either the arm fragment's telomere or the partner's telomere (LaFountain et al., 2002). Thus, ablating telomeres also inactivates tethers. We did similar experiments in CalA-treated cells. In four cells treated with CalA we cut the telomeres toward which the backwards moving chromosomes were moving. In all four cells cutting the telomere caused backwards movements to stop (Figure 3). In our experiments, in two of the cells the backwards movements stopped and no further movements ensued (Figure 12E). In the other two cells the moving chromosomes reversed directions and moved toward their original poles. These experiments disabled the tethers in a second way and confirm that tethers pull the chromosomes backwards. Thus, tether elasticity is preserved when PP1 is blocked during anaphase.

The previous results all indicate that the backwards movements caused by CalA are due to tethers that remain elastic throughout anaphase, and that inhibiting PP1 causes the tethers to remain elastic. Our results, illustrated in cartoons in Figure 12, are: Cutting tethers stops the backwards movements (Figure 12B); cutting chromosome arms from backwards-moving chromosomes stops the backwards movements (Figure 12C); cutting chromosome arms before the backwards movements start prevents subsequent backwards movements (Figure 12D); and ablating a telomere of a backward-moving chromosome stops the backwards movements (Figure 12E). These results lead to the conclusion that in CalA-treated cells tethers are elastic and cause the backwards movements. This conclusion can be tested further and elaborated on by comparing arm fragments produced in anaphase in control cells vs. those produced in CalA-treated cells. Such comparisons could answer several questions. If our conclusion is correct, arm fragments in CalA-treated cells should move all the way to their partner throughout anaphase, independent of tether length, whereas those in control cells move to their partners only in early anaphase, when tether lengths are short (LaFountain et al., 2002). This comparison directly tests our conclusion. The data will also indicate whether arm-fragment speeds are increased when PP1 is blocked. This would occur if phosphorylation continues throughout anaphase, assuming that tethers become hyper-phosphorylated and that elasticity (and arm-fragment velocity) increases when tethers are hyper-phosphorylated, similar to increased force produced by hyper-phosphorylated myosin (Sheykhani et al., 2013).

We now describe comparisons between arm-fragment movements in control cells vs. in CalA-treated cells.

### Comparisons of Arm Fragments in CalA-Treated Cells vs. in Control Cells

We compared arm-fragment movements in CalA-treated cells with those in control cells in order to further test whether CalA preserved tether elasticity. LaFountain et al. (2002) concluded that tether elasticity decreases as tether length increases, because at longer tether lengths arm fragments move shorter distances, at reduced speeds. Since tethers persist even when arm fragments do not move (Forer et al., 2017), long tethers remain attached to separating chromosomes but are inelastic. If CalA prevents loss of tether elasticity, as we concluded, when cells are treated with CalA at the start of anaphase, arm fragments produced later in anaphase, with longer tethers should have elastic tethers, unlike those in control cells. To test our interpretation, therefore, we used arm-fragment movements as a measure of tether elasticity and compared movements in control cells vs. those in CalA-treated cells. We measured the control cell parameters ourselves to verify the original conclusions by LaFountain et al. (2002), and to assure ourselves that differences in methodology employed in the two sets of experiments do not influence the results.

#### General Description of Arm Fragment Movements and of Our Methods

**Figure 4:**
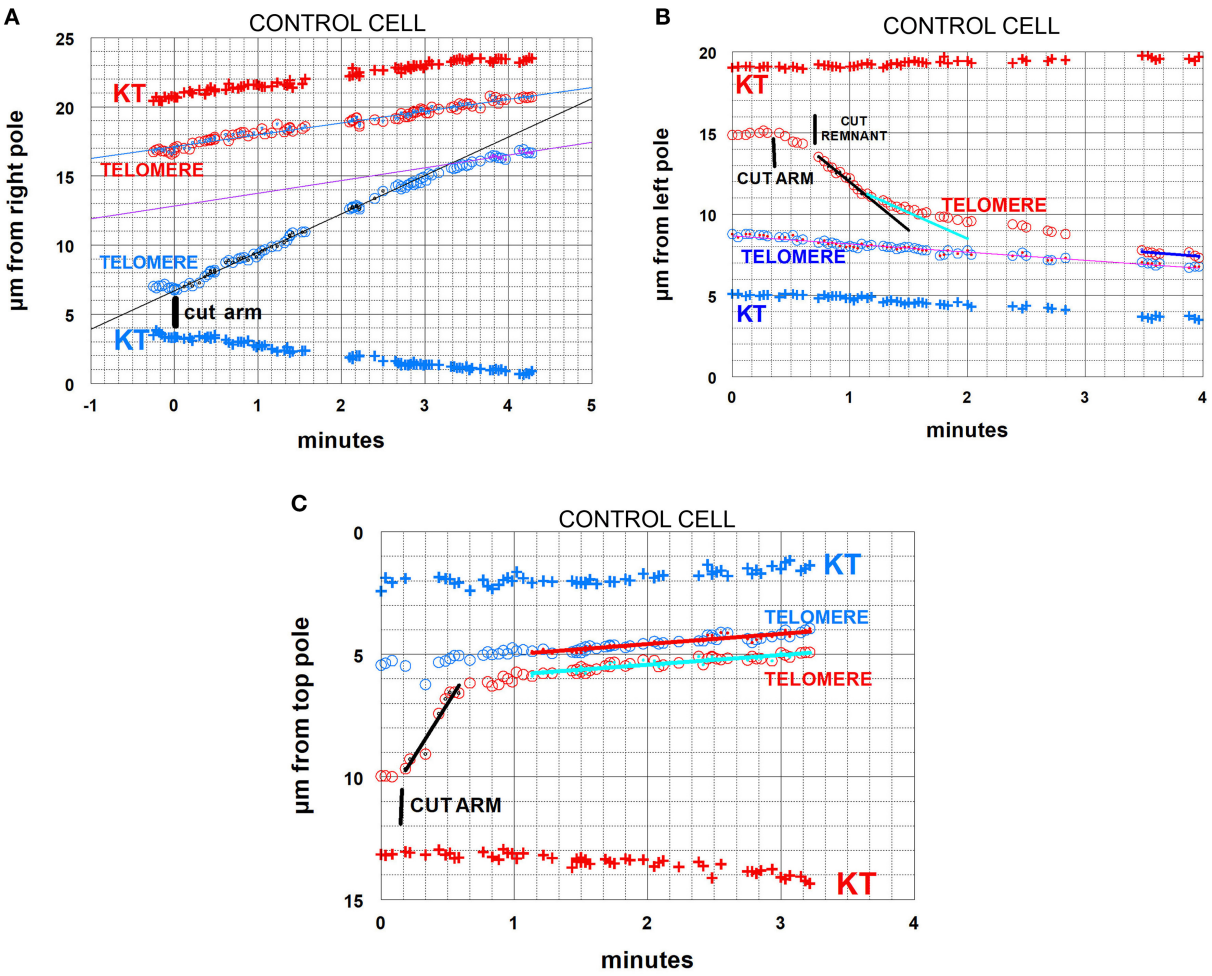
Control cells. Arm-fragment movements in three different cells. In all three graphs the positions are plotted vs. a fixed position at a pole. In these and all subsequent graphs, kinetochore positions are labeled KT. Telomere positions are labeled as such; those moving to one pole are red, those to the other pole blue. The drawn lines are lines of best fit to the tiny points in the larger symbols indicated in the same color as the lines. **(A)** illustrates an arm fragment (blue circles) that moves with constant speed toward the opposite telomere (red circles), then stops moving to the opposite telomere (at about 3 min 20 s) but continues to move toward the opposite pole at the same speed as the opposite telomere, as if being towed by that telomere. The arm was cut when the two telomeres were about 10 μm apart. The arm fragment stopped moving toward the opposite telomere when it was about 4 μm away from that telomere, so in this cell the arm fragment moved backwards (because of tether shortening) by about 6 μm out of the original 10 μm. The lines through the indicated points (inserts in the circles in the same colors as the lines) are lines-of-best-fit as determined by the computer program. **(B)** illustrates an arm fragment (red circles) that moves with varying speeds toward the opposite telomere (blue circles), faster at first (black line), then slower, at about 1 min:20 s (sky-blue line), and gradually slower until it stops moving toward the opposite telomere (at about 3 min 30 s on the graph), after which it moves to the opposite pole together with (and at the same speed as) the opposite telomere, as if being towed by that telomere. The arm was cut when the telomeres were about 6.5 μm apart, and the arm fragment stopped moving when it was about 0.5 μm from the opposite telomere, so in this cell the arm fragment moved backwards (because of tether shortening) by about 6 μm out of the original 6.5 μm. The arm fragment seemed to be moving slowly immediately after the arm was cut, so (at about 40 s on the graph) we cut the region between the fragment and the amputated arm to cut the “remnant,” after which the fragment sped up. **(C)** illustrates a cell in which the arm fragment (red circles) moves backwards with one speed (black line). It stopped moving toward the opposite telomere when it was about 1 μm away from it (at about 40 s on the graph), but then moved to the opposite pole with the same speed as the opposite telomere, maintaining a constant distance from it, as if towed by the opposite telomere. In this cell the arm was cut when the telomeres were separated by about 4.5 μm, the arm fragment moved until it was 1 μm from the opposite telomere, so in this cell the arm fragment moved backwards (because of tether shortening) by about 3.5 μm out of the original 4.5 μm.

After an arm fragment is formed, that arm fragment's telomere moves toward its partner's telomere and both move toward the same pole. Measuring the distances between moving telomeres does not give an accurate impression of speeds or distances moved. One needs to plot each of their positions in space in order to measure actual speeds and distances the fragment moved. We chose a reference point in each cell, either near a pole or near the equator, and we measured the kinetochore and/or telomere distances that from the fixed point. The fixed points were chosen so that the telomeres and kinetochores in question move in a relatively straight line from or toward the fixed point. After converting pixel distances to distances in the cell, the points were plotted on graphs of distance vs. time. As seen from the graphs, arm fragments sometimes move to (or toward) their partners with one speed (e.g., Figure 4A), but sometimes they move with variable speeds, slowing as they get closer to their partner (e.g., Figure 4B). From graphs of this kind we obtained arm-fragment speeds and distances moved. *Speeds* were determined from the slopes of the lines of distance vs. time; for movements that slowed down, we used only the initial (higher) velocities. The *distances* that the arm fragments moved were also determined from the graphs. It was not as simple as determining the distance between start and end points of arm fragment motion, however. In many cases, the arm fragment stopped moving toward the partner telomere after moving a certain distance, but then continued to move at the same speed as the partner telomere. The arm fragment remained at a constant distance from the other telomere, as if it was being towed by something attached to the moving telomere (Figures 4A–C). Since the aim of measuring the distance that arm fragments moved is to determine the extent by which the arm fragment's initial tether shortened (contracted), we first determined from the graphs the distance between the two telomeres at the time the arm was severed. Then we determined the closest distance between the two telomeres that the arm fragment moved to. The difference between the two indicates how much the tether shortened (illustrated in the legend to Figure 4). In collecting data, we tabulated distances moved and tether lengths for each cut arm and then converted the distance values to fractions of the tether length at the time the arm was cut.

There is a potential complication for measurements of both speeds and distances moved: even when the severing of the arm appears complete and the arm fragment rapidly moves backwards to the partner telomere, sometimes “invisible” contractile material still connects the arm fragment to the amputated arm. This was demonstrated in experiments in which tethers of moving arm fragments were cut, after which the arm fragments moved backwards toward the amputated arm (Sheykhani et al., 2017; Forer et al., 2018). In the present experiments, to ensure that chromosome “remnants” did not retard the speed or shorten the distance that arm fragments moved, we often cut behind the moving arm fragment, especially when the fragments slowed down or appeared to stop before reaching the partner (Figure 4B).

**Figure 5:**
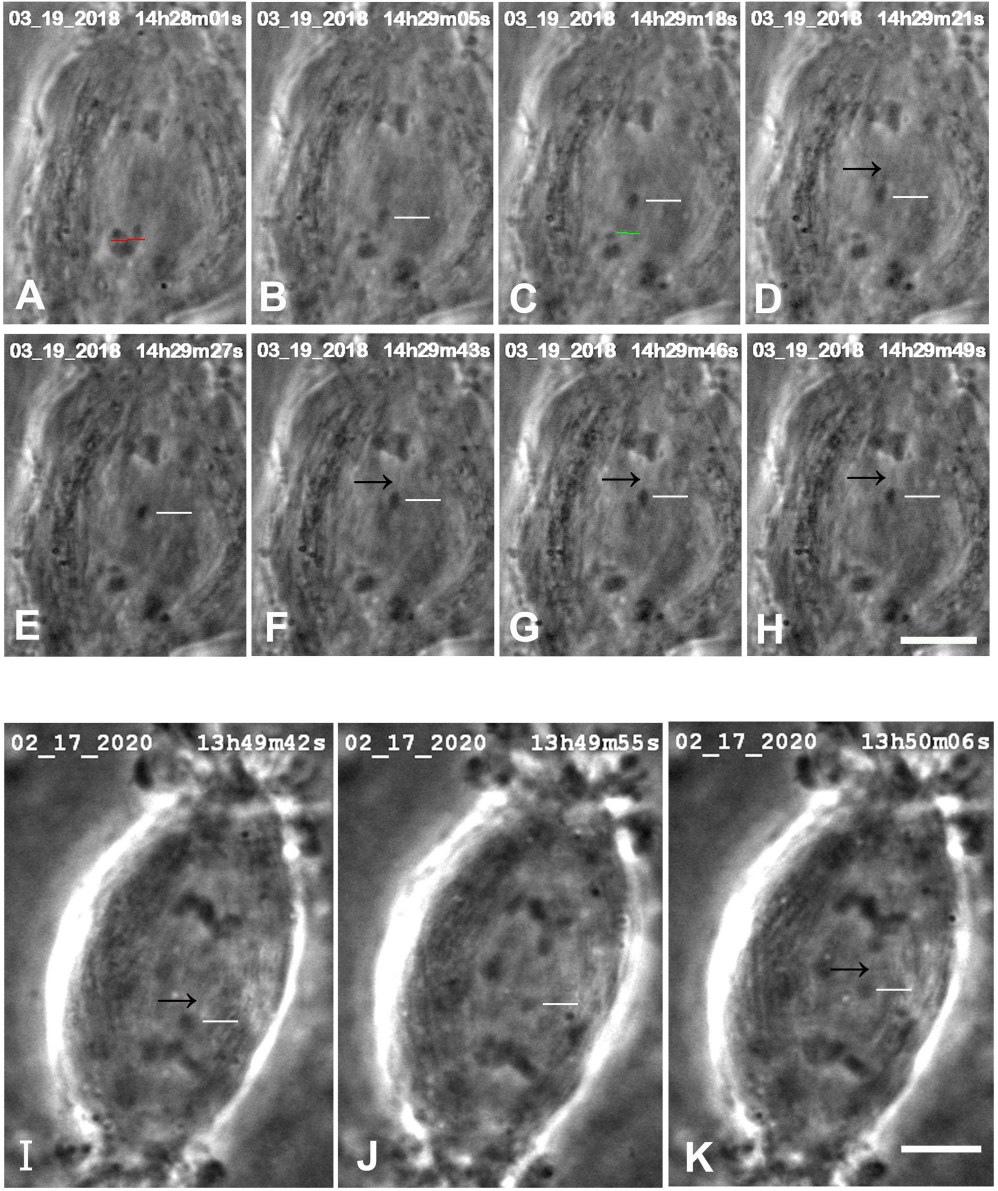
Control cells in which images were seen that might represent tethers. (A–H) are from one cell, (I–K) are another**. **In ***the top cell***, arms were cut from an anaphase chromosome (at the position of the red line in A), after which a resultant arm fragment (indicated by the white horizontal lines in B–H) moved toward its partner chromosome. (The green line in C is the position at which the possible “remnant” was cut.) In some of the images faint black lines extend between the telomeres of the arm fragments and those of their partners, pointed to by black arrows in (D–H). The scale bar in (H) represent 5 μm. In ***the bottom cell***, the arm fragment moves toward its partner, as indicated by horizontal white lines in (I–K). In images (I,K) faint black lines extend from the telomere of the arm fragment toward or to that of its partner, pointed to by the black arrows. The scale bar in (K) represents 5 μm.

While not directly related to our experimental protocol, it is worth noting that in images in several of the sequences there were faint dark “lines” (against the lighter spindle background) in positions where one expects to see tethers. These presumed tethers were not seen in all cells, and when seen were not seen in every image (e.g., Figure 3A,F,G, and the two cells in Figure 5). But they were seen often enough for us to venture that they are images of tethers.

**Figure 6:**
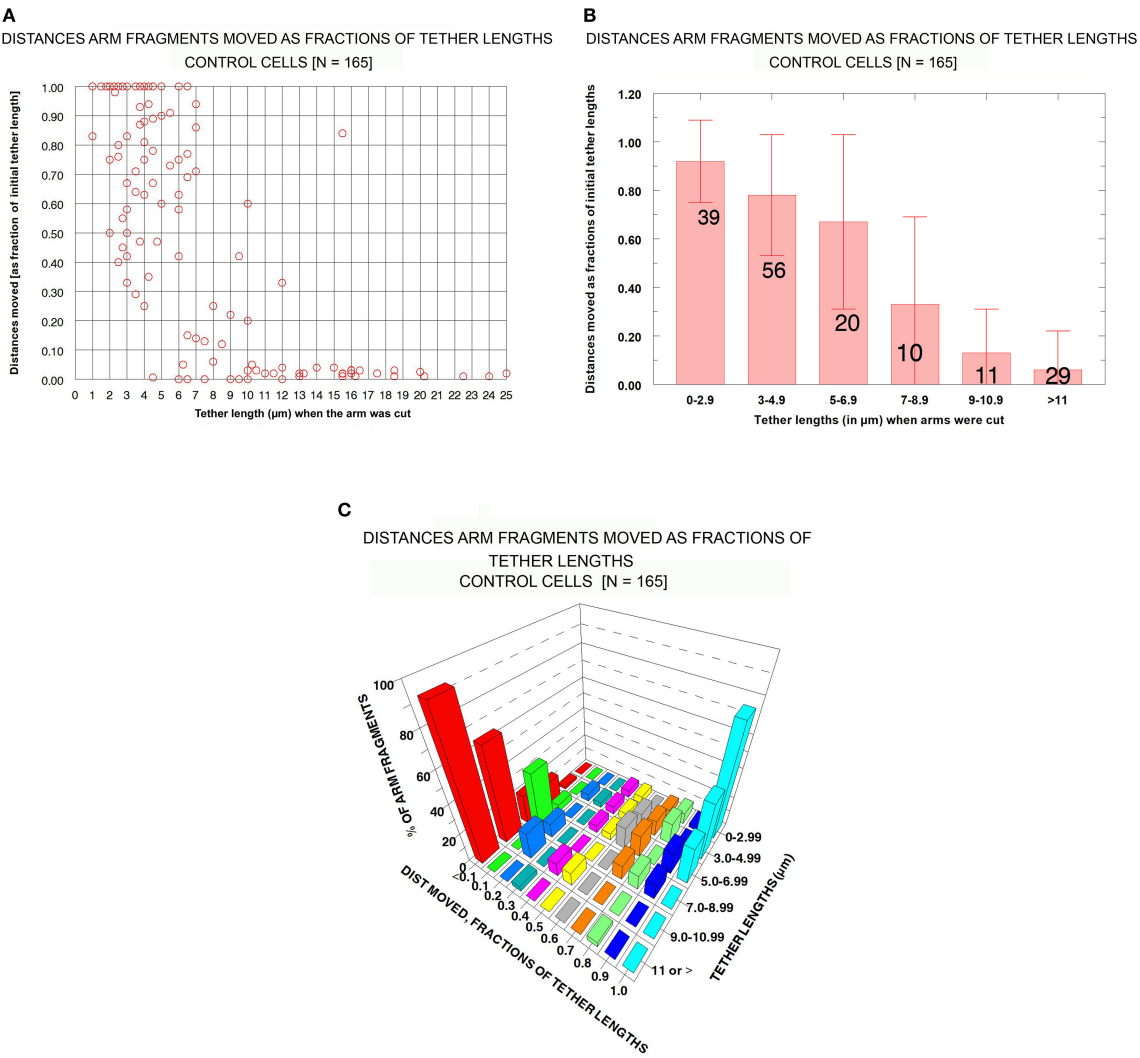
Control cells: Distances moved by arm fragments (as fractions of their initial tether lengths) at different initial tether lengths. **(A)** is a scatter diagram of all 165 arm fragments. Distance moved (as fraction of the original tether length) is plotted on the ordinate vs. tether length at the time the arm was cut (plotted on the abscissa). **(B)** contains all the individual points from **(A)** grouped into tether lengths; the ordinate represents the average fractional distances with standard deviations (brackets). The numbers of arm fragments in each group are indicated on each bar. **(C)** contains the same data, grouped, averaged, and plotted so one can visualize in the same graph the percentage of arm fragments at grouped tether lengths that moved specific fractions of tether lengths at different tether lengths.

***Control cells****: Distances moved by arm fragments*. The question we want to ask is: how elastic are tethers of different tether lengths? That is, how much do tethers contract when free to do so (i.e., when an arm fragment is released from the rest of the chromosome)? In our assay we measure tether lengths when the arm is cut, measure the distance moved by the arm fragment, and convert the actual distance to the fractional distance (of the initial tether length) that the arm fragment moved. The result is the fraction (%) that the tether contracted at that tether length. We measured these parameters for our entire sample of control cells (*N* = 165) encompassing initial tether lengths of ~1 μm to >11 μm. The results (fractional distances moved vs. initial tether length) for the sample of 165 cells indicate that (1) as tether lengths increase, tethers shorten less and less of their initial lengths, and (2) longer tethers are inelastic in that arm fragments produced from them do not move. These results are illustrated graphically in Figure 6 in a scatter diagram (Figure 6A), in a bar graph (Figure 6B), and in a 3-D plot to show several parameters at once (Figure 6C). Many tethers 1–3 μm long shortened 100% of their initial length (Figures 6A,B) while most tethers > 11 μm did not shorten at all or shortened by 4% at most (corresponding to 0.3–0.4 μm). Thus, shorter tethers are completely elastic, longer tethers are not elastic at all, and intermediate length tethers are in between, with a trend of less and less elasticity as tethers elongate (Figure 6B).

***Control cells****: Speeds of arm fragment movements*. We measured velocities of all 165 arm fragments. Comparisons of velocities at different tether lengths may give information about how tethers change as they lengthen during anaphase. For example, if tether dephosphorylation leading to inelasticity is gradual along the entire length of the tether, arm-fragment movements might gradually slow as tethers become longer. Or if dephosphorylation is complete at the ends of tethers and then gradually moves inwards toward the middle, arm-fragment speeds would not change but the arm fragments would move shorter distances.

In our analysis we omitted those arm fragments that had zero velocity (as shown in Figure 6A). This was done in order to consider only those movements due to tether elasticity. An additional group of fragments was eliminated from the data because of another consideration: the likelihood that some of the movement of very small distances is not due to tether elasticity. When tethers are cut between separating anaphase chromosomes, each of the previously attached chromosome arms shortens/contracts by ~10% of its length (Forer et al., 2018). Similarly, when an arm is severed to form an arm fragment, the opposite arm contracts because there is no longer tension from the tether (Forer et al., 2018). We are concerned that when tethers are inelastic and we sever arms, the arm fragment might be propelled backwards, momentarily, as the arm of the partner chromosome contracts when tension is removed. We think that this is why small, brief, sometimes rapid arm-fragment movements took place, resulting in arm fragments that moved very short distances. None of the arm fragments that moved <10% of the initial tether length shown in Figure 6A moved as much as 0.5 μm, so we assumed that those brief short movements were not due to tether elasticity. Thus, we omitted from our analysis all movements that were <10% of the initial tether length. After eliminating those arm fragments from consideration, we were left with 125 arm fragments. Our analysis indicated that, contrary to the findings of LaFountain et al. (2002) and to our expectations, arm fragments did not move slower when the initial tethers were longer.

**Figure 7:**
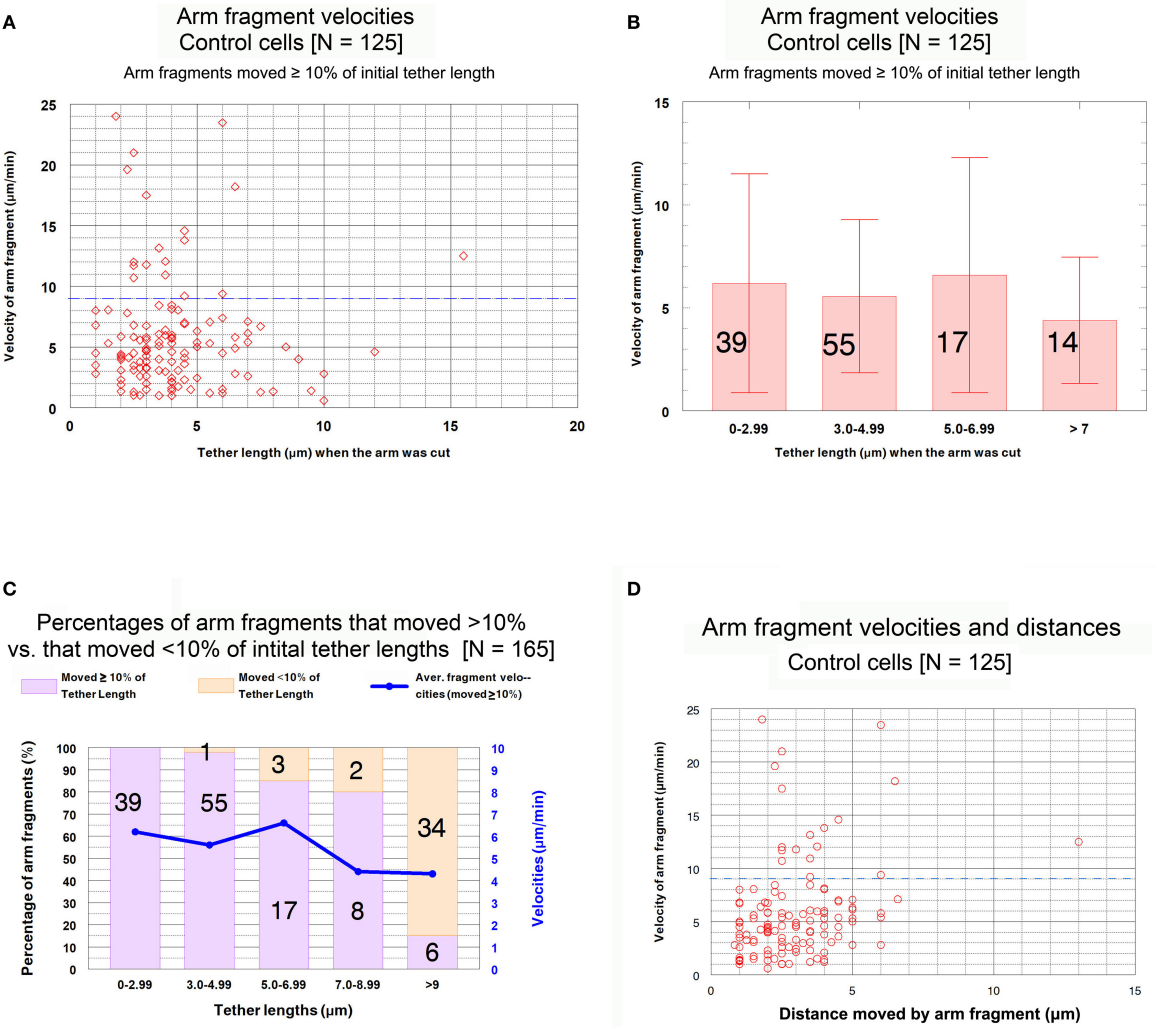
Control cells. Velocities of arm fragments at different tether lengths and numbers of arm fragments that moved >10% and <10% of the initial tether lengths. **(A)** is a scatter diagram of the velocities of the 125 arm fragments that moved ≥10% of their initial tether length, plotted on the ordinate, vs. tether length at the time the arm was cut, plotted on the abscissa. There is a dashed blue line across the graph at a velocity of 9 μm/min to illustrate that a large majority of the arm fragments (107/125) moved with velocities <9 μm/min. **(B)** contains all the individual points from **(A)** grouped into tether lengths; the average velocities are plotted together with standard deviations (brackets); numbers in each groups are indicated on each bar. We chose the ranges into which tether lengths were grouped in order to obtain reasonable numbers per group for the longer tether lengths. The averages are not statistically significantly different (using Student's *t*-test) so there is no trend. **(C)** contains the entire set of 165 arm fragments, grouped into specific tether length ranges (abscissa), showing the percentages (left ordinate) of the 125 arm fragments that moved ≥10% of the initial tether length (violet bars) and the percentages of those that moved <10% of the initial tether length (rose bars), as well as illustrating the average fragment velocities (right ordinate) of the 125 arm fragments that moved ≥10% of the initial tether length in each of the tether length ranges. The numbers on each bar represent the number of arm fragments in that range of tether lengths. **(D)** is a scatter diagram of the velocities of arm fragments that moved >10% of the initial tether length (ordinate) vs. the actual distance in the cell that each arm fragment moved (abscissa). There seems to be no trend. The dashed blue horizontal line at 9 μm/min illustrates that most arm fragments moved with velocities of <9 μm/min.

Our data with respect to this conclusion are presented in Figure 7. The scatter diagram in Figure 7A shows that there is no trend in arm-fragment velocity (ordinate) with initial tether length (abscissa), and that most (85%, 107/125) arm fragments had velocities of <9 μm/min (dashed blue line). Those that moved faster were spread throughout all tether lengths. The same data presented as a bar graph within grouped lengths (the brackets indicate standard deviations), Figure 7B, also indicate that there is no trend. The graph in Figure 7C shows how many arm fragments fit the two classes, movement >10% and movement <10% of the initial tether lengths, as well as indicating the average velocities shown in Figure 7B. We conclude from these data that arm-fragment velocities do not change as initial tether lengths increase. Nor do arm-fragment velocities vary with the actual distances that the arm fragments move (Figure 7D). The relationships between arm-fragment velocity and initial tether length, or between velocity and actual distance moved, however, are not main issues we want to address.

**Figure 8:**
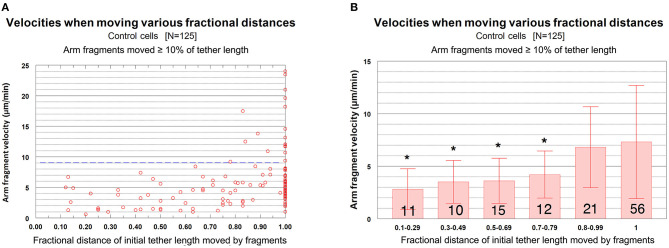
Control cells. Velocities of arm fragments vs. fractional distances of the initial tether lengths that the fragments moved, for all fragments that moved ≥10% of the initial tether length. **(A)** is a scatter diagram of all 125 arm fragments in our sample, fragment velocity on the ordinate and distance moved (as a fraction of the original tether length) on the abscissa. The dashed blue line at a velocity of 9 μm/min illustrates that a large majority of the arm fragments (107/125) moved with velocities <9 μm/min. Of the 18 arm fragments that moved with velocities ≥9 μm/min, none moved less than 0.8 of the length of the tether and only five moved less than the complete length of the tether. **(B)** contains the same data as in **(A)**, except the points were grouped into ranges of fractional tether lengths; the bars represent average velocities and bracketed lines represent standard deviations. The numbers on each bar represent the numbers of arm fragments in that group. The two groups with highest fractional distances, [0.8–0.99] and [1], are not significantly different from each other (using Student's *t*-test) but both are statistically significantly different from the four other groups (indicated by the asterisks).Each individual comparison with each of the two groups of fractional distances [0.8–0.99] and [1] have *t*-test probabilities of <0.004 for being from the same distribution except for the comparison of the [0.7–0.79] group with the [0.8–0.99] group for which the probability of being from the same distribution is ~0.02.

The question we want to ask is whether the velocities of arm-fragment movements depend on how much the tether shortens, i.e., on the fractional distances of the initial tether lengths that arm-fragments move. That is, were speeds different for fragments whose tethers completely shortened compared with those whose tethers incompletely shortened? This might give clues about how and whether dephosphorylation affects elasticity. Accordingly, we plotted arm-fragment velocities vs. fractional tether length distances that the fragments moved as a scatter diagram (Figure 8A) and as a bar graph after grouping the individual points into ranges and plotting averages and standard deviations (Figure 8B). These graphs illustrate that arm fragments moved faster, on average, when their tethers completely shortened than when their tethers incompletely shortened (Figure 8B). All 18 arm fragments that moved with velocities >9 μm/min (blue dashed line in Figure 8A) moved ≥ 80% of the length of the initial tether. Over 70% (13/18) moved the complete length of the initial tether (i.e., the tether completely contracted). All fragments in which tethers shortened by <80% of their initial length moved at speeds below 9 μm/min (Figure 8A). When the data are incorporated into ranges and averaged, the velocities of arm fragments that moved <80% of the initial tether length are statistically significantly different from those that moved >80% of their initial tether length (Figure 8B). Those arm fragments for which the tethers shortened ≥80% of the initial length, the only arm fragments that moved at speeds above 9 μm/min, moved with maximum speeds of up to 24 μm/min (Figure 8A).

We conclude that arm-fragment velocities do not vary with initial tether length *per se*, but rather depend on how much their tether contracts/shorten.

To investigate which parameters of arm-fragment movements change when prevention of dephosphorylation keeps the tethers elastic, we now compare these baseline parameters in control cells with the same parameters obtained from cells treated with Cal-A at the start of anaphase. We analyzed the data from cells treated with CalA in the same manner as we analyzed those from control cells.

**Figure 9:**
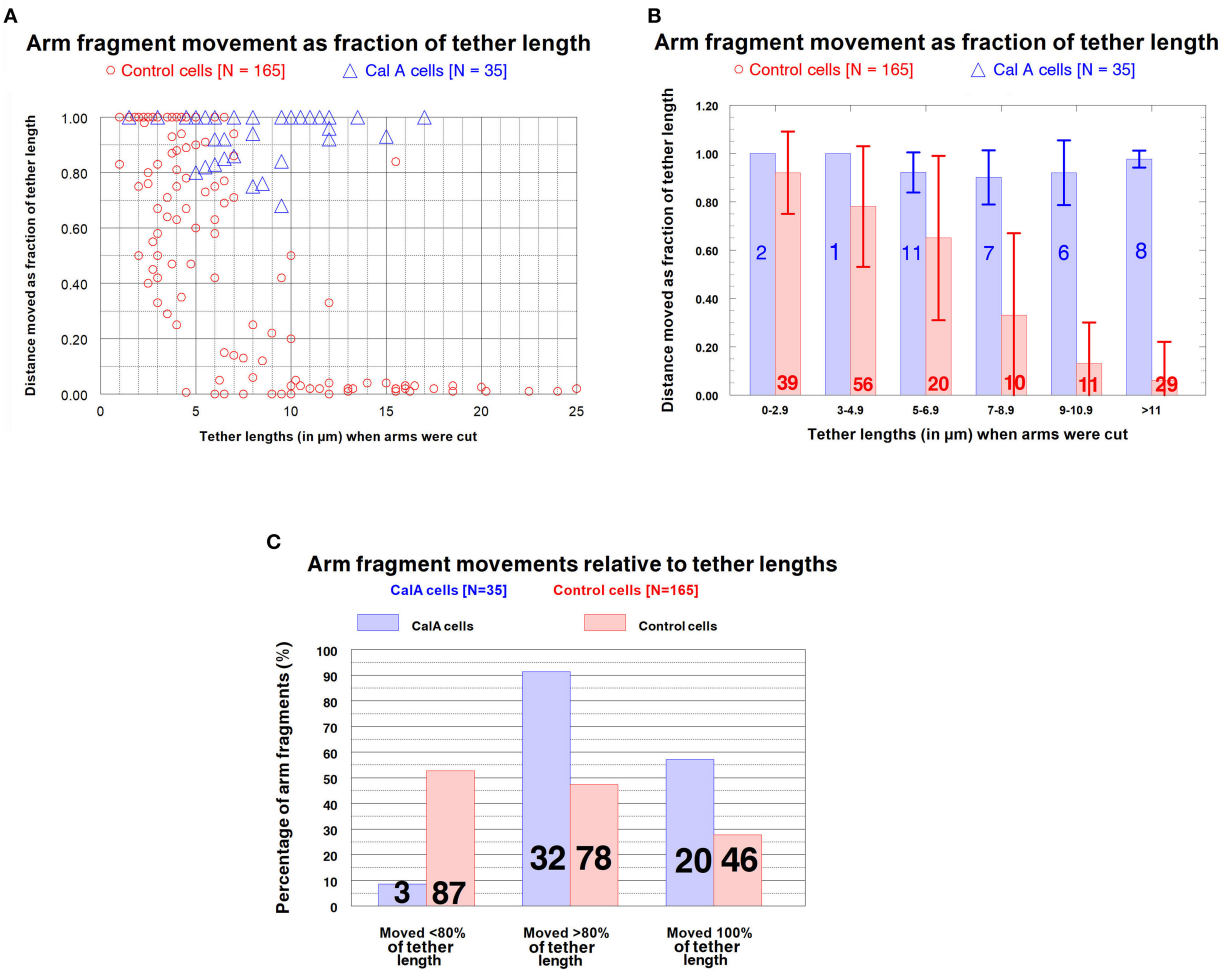
CalA-treated cells (blue) compared with control cells (red). **(A,B)** Distances moved by arm fragments (as fractions of initial tether lengths) at different tether lengths. **(A)** is a scatter diagram of arm fragments from control cells (red) and CalA cells (blue), plotted on the ordinate as distance moved (as fraction of the original tether length) vs. tether length at the time the arm was cut, plotted on the abscissa. (The data for control cells are also presented in Figure 6A.) At tether lengths longer than 7 μm the arm fragments in CalA cells moved longer distances than those in control cells; only three of the arm fragments in CalA cells moved less than 80% of the length of its tether. **(B)** compares the control cells (red) with CalA cells (blue) after the values from **(A)** were grouped into varying tether lengths (abscissa), averaged (bars), and standard deviations calculated (brackets). The numbers on each bar represent the number of arm fragments in that group. (The data from control cells were presented earlier in Figure 6B.) Arm fragments in CalA cells moved longer distances (as fractions of the initial tether lengths) than those in control cells: using Student's *t*-test, the probabilities that the blue bars (CalA cells) are from the same distributions as the red bars (control cells) at the same tether lengths, starting from tether lengths 5–6.9 and moving to the right, are *p* ~ 0.002, *p* < 0.005, *p* < 0.005, and *p* < < 0.005. (**C)** illustrates graphically the frequencies (ordinate) with which arm fragments move different fractional distances of the initial tether lengths (abscissa) in control cells (red) and CalA-treated cells (blue). The numbers on each bar represent the number of arm fragments in that group. Almost all arm fragments in CalA-treated cells moved >80% of the tether length, at tether lengths up to 17 μm **(A)**, whereas less than half those in control cells did.

***Cells treated with CalA:****Distances moved by arm fragments*. Arm fragments in CalA-treated cells rarely (3/25 fragments) moved less than 80% of the original tether length (Figure 9C). Most (120/135) moved 100% of the original tether length (i.e., completely to the opposite telomere) over a variety of tether lengths, up to 17 μm (Figures 9A,C), considerably different from control cells (Figures 9A–C). Thus, treatment with CalA causes tethers to remain elastic throughout anaphase.

**Figure 10:**
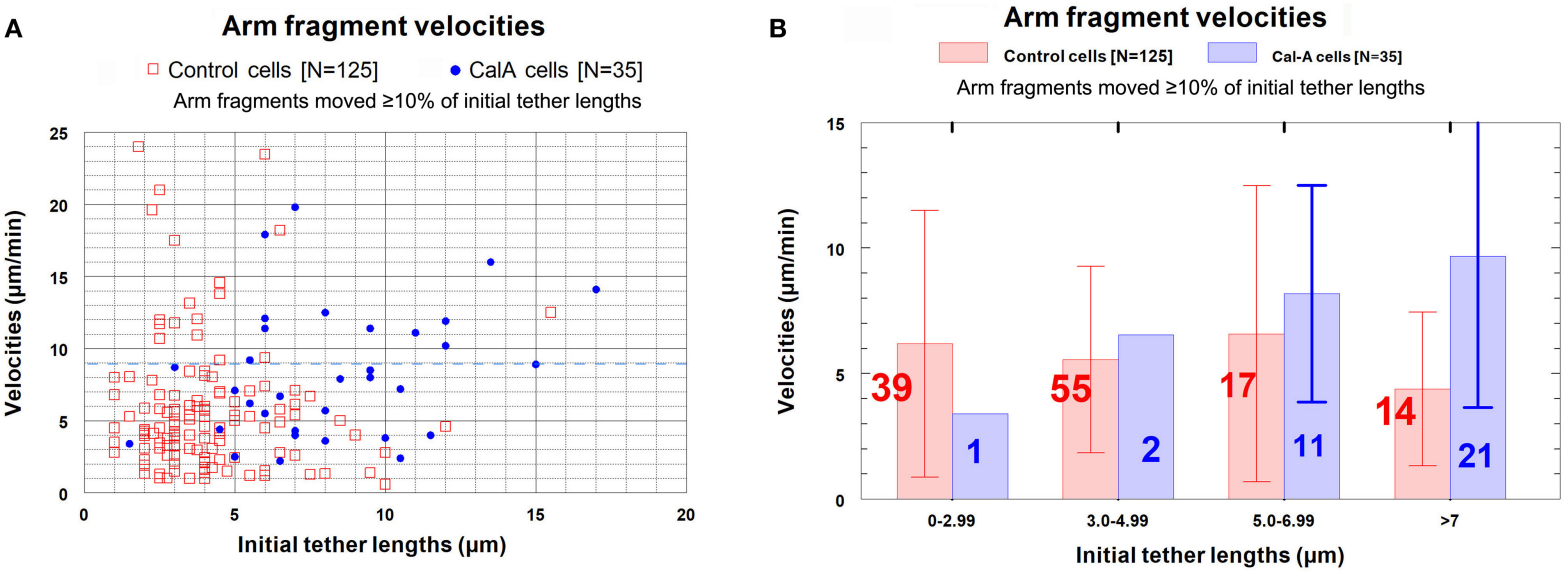
Arm fragment velocities at different tether lengths in control cells compared with those in CalA cells. (The data from control cells was presented in Figure 7A.) **(A)** is a scatter diagram showing all arm fragments in control cells (red squares) and all arm fragments in CalA cells (blue dots). A dashed blue line at velocity of 9 μm/min illustrates that most arm fragments in control cells (107/125) and in CalA-treated cells (22/35) moved with velocities ≤ 9 μm/min. **(B)** contains the points in **(A)** grouped into tether length ranges; the average velocities in each group (ordinate) are indicated by the vertical lengths of the corresponding bars, and the number in each group is indicated on each bar. The red bars and numbers are for control cells and the blue bars and numbers are for Cal-A cells. The brackets represent standard deviations.

**Figure 11:**
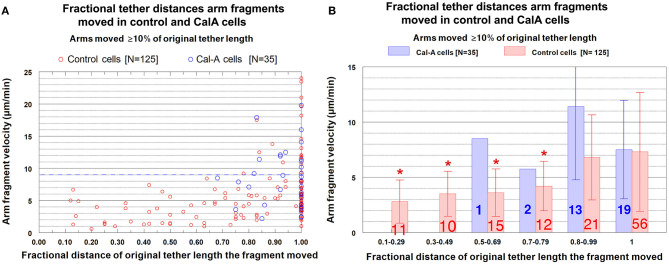
Arm fragment velocities when moving different fractional distances of the initial tether lengths, comparing control cells with CalA-treated cells. **(A)** is a scatter diagram, for all arm fragments that moved ≥10% of the initial tether length, of the velocities, plotted on the ordinate, vs. distance moved (as a fraction of the original tether length), plotted on the abscissa. Each arm fragment in control cells is indicated by a red circle, in CalA cells by a blue circle. (The control cell data were presented in Figure 8A.) The dashed blue line across the graph at a velocity of 9 μm/min illustrates that a majority of the arm fragments in control cells (107/125) and in CalA cells (22/35) moved with velocities <9 μm/min. **(B)** contains the same data as in **(A)**, with the points grouped into ranges of fractional tether lengths; the average values and standard deviations (bracketed lines) are presented. The numbers on each bar represent the numbers of arm fragments in that group, the velocities are presented on the ordinate, and the asterisks represent statistically significant differences from those without asterisks, as explained in the legend to Figure 8B.

***Cells treated with CalA:****Velocities of arm fragment movements*. As in control cells, arm fragment velocities in CalA-treated cells do not seem to depend on original tether length (Figure 10); using Students *t*-test, the grouped values in Figure 10B for CalA cells are no significantly different from each other. Arm-fragment velocities in CalA are not different from those in control cells, except that there are proportionally more arm fragments that moved faster in Cal-A cells at longer tether lengths than in control cells (Figure 10). In addition, a larger fraction of arm fragments in CalA cells moved ≥ 80% of the initial tether length than in control cells (Figure 11). Thus, treatment with CalA does not cause arm fragments to move faster, i.e., does not increase tether elasticity.

We conclude that backwards movements caused by CalA are due to elastic tethers. CalA causes tethers to stay elastic for longer times (longer tether lengths) during anaphase, consistently shortening by 80–100% of the original tether length, but CalA does not cause the speeds to increase above those of control cells. Thus, inhibiting PP1 at the start of anaphase prevents loss of tether elasticity during anaphase but does not cause increase in tether elasticity.

## Discussion

**Figure 12:**
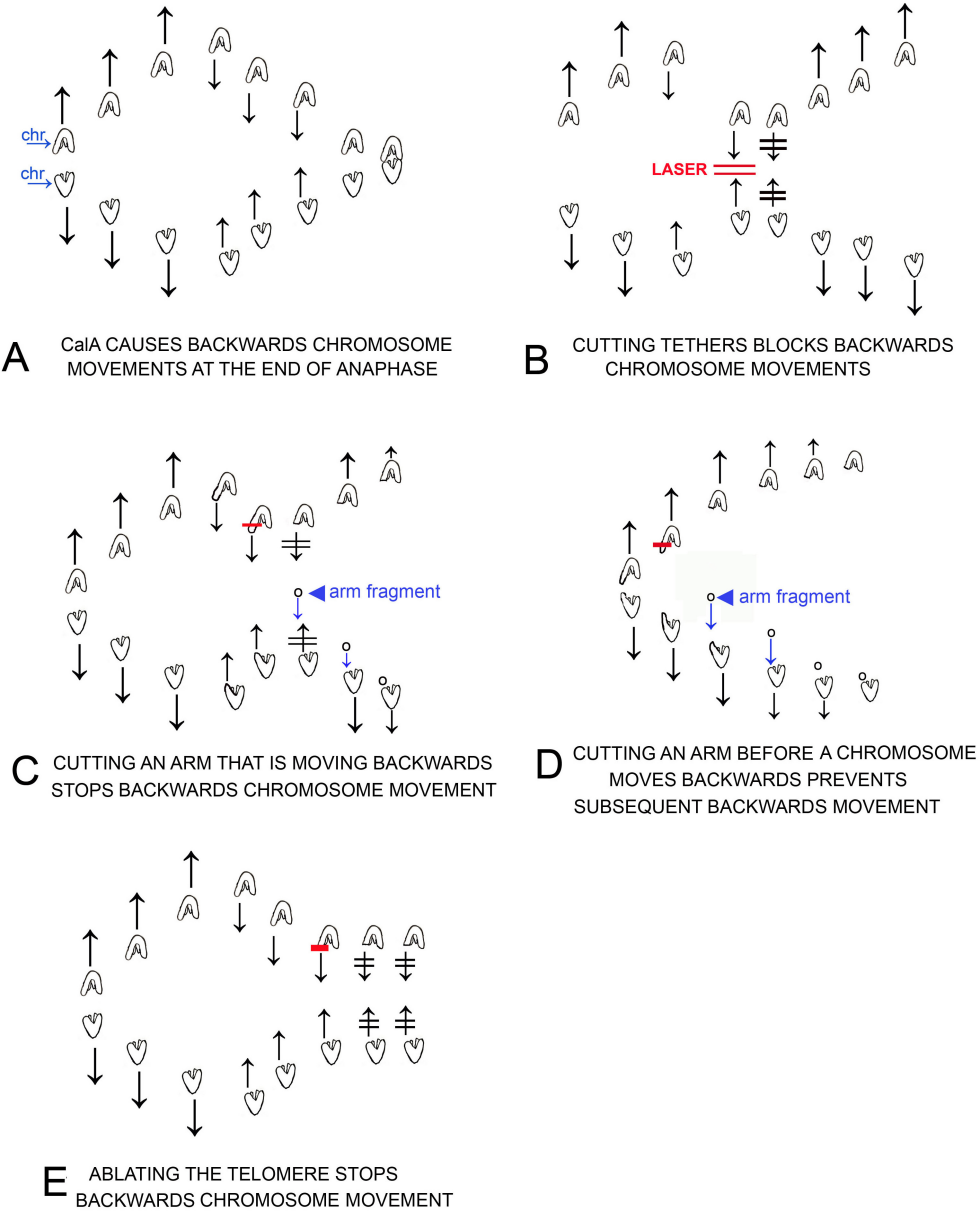
Cartoons that illustrate individual experiments on cells treated in early anaphase with CalA, results described in the text. Chromosomes are drawn as indicated in **(A)**, in which separating chromosomes are labeled chr. Laser cuts are indicated by red lines, e.g., as labeled in **(B)**. Arm fragments are pointed to with arrowheads as in **(C,D)**. Arrows represent directions of motion of the associated chromosomes; two lines through arrows indicates that chromosome motion stopped, e.g., after laser irradiation in **(B)**. **(A)**: CalA treatment causes chromosomes to move backwards after they move to the spindle poles. **(B)** illustrates that cutting the tethers connected to backwards moving chromosomes stops the backwards motion and often the chromosomes reverse directions and move again to their original poles. **(C)** illustrates that cutting an arm of backwards moving chromosomes stops the backwards motion and often the chromosomes reverse directions and move again to their original poles. The arm fragment that is formed moves to the opposite chromosome as usual. **(D)** illustrates that cutting an arm from early anaphase chromosomes, before the chromosomes reach the pole, prevents the chromosomes from moving backwards after they reach the pole. **(E)** illustrates that cutting/ablating telomeres stops the backwards movement of the partner chromosomes.

The major conclusions from our experiments are (1) that inhibition of PP1 by CalA causes tethers to remain elastic throughout anaphase instead of becoming inelastic in later anaphase; (2) that elastic tethers pull chromosomes backwards toward each other at the end of anaphase after pole-directed forces weaken; and (3) that inhibition of PP1 preserves tether elasticity but does not increase tether elasticity (i.e., arm-fragment speeds do not increase). The evidence supporting our conclusions are strong (Figure 12). Cutting tethers stops backwards movements (Figure 2), cutting arms that lead the backwards moving chromosomes stops their backwards movement (Figure 3), cutting arms from chromosomes before the chromosomes reach the poles stops subsequent backwards movements (Table 2), ablating the telomere of the arm to which backwards chromosomes are moving stops the backwards movements (Figure 4), and finally, tethers in CalA cells are elastic throughout 80–100% of their length, for all tether lengths up to 17 μm, maintaining elasticity at lengths much longer than tethers in control cells remain elastic (Figure 9). Thus, inhibition of PP1 by CalA causes tethers to remain elastic throughout anaphase, and the CalA phenotype arises because elastic tethers pull chromosomes backwards at the end of anaphase.

We suggest that in control cells phosphorylated tethers appear in early anaphase and they become dephosphorylated later in anaphase. While our data indicate that tethers lose elasticity when some component is dephosphorylated by PP1, we have not proved that tethers themselves are phosphorylated or that PP1 acts directly on them. That is our working hypothesis, though. This hypothesis is consistent with titin being a component of tethers. Titin, the giant elastic protein responsible for elasticity in heart muscle (Kruger and Linke, 2006; Hidalgo and Granzier, 2013; Hamdani et al., 2017) and skeletal muscle (Linke et al., 1998; Tskhovrebova and Trinick, 2003), connects (is present between) the telomeres of separating anaphase chromosomes (Fabian et al., 2007a,b). Titin elasticity is a function of its phosphorylation state [titin is elastic when phosphorylated in the PEVK region and is inelastic when dephosphorylated (Hidalgo et al., 2009)]; titin phosphorylation (in the PEVK region) is removed by PP1 (Hidalgo et al., 2009). Our hypothesis is also consistent with the localization and activity of PP1 during mitosis: PP1 is active during anaphase and is present in the interzone (the region between separating anaphase chromosomes, where tethers are) (Trinkle-Mulcahy et al., 2003). PP1 also associates during mitosis with both chromosomes and spindle microtubules (Andreassen et al., 1998).

Other experiments also suggest that tethers themselves are phosphorylated. In cells in which arm fragments stop moving before they reach the partner telomere, those arm fragments are pulled toward the pole at a constant distance from the moving telomere (Figure 4). It seems most likely that the arm fragment is attached to the partner telomere by an inelastic portion of the tether and that the inelastic tether pulls the arm fragment as the unsevered chromosome moves to the pole. Since the arm fragments start being pulled at different distances from the partner telomeres (e.g., Figures 4A,C), this implies that tethers do not become inelastic along their entire length all at once but rather become inelastic gradually, at different positions along their length. This is most easily explained if the tethers themselves start anaphase completely phosphorylated and then lose phosphorylation along their length during anaphase. Kite and Forer (2020) discussed three possible scenarios in which this might happen: tethers lose phosphorylation from the middle outwards, or lose phosphorylation evenly along the length, or lose phosphorylation from the ends inwards. Since our experiments indicate that an inelastic length of tether remains after maximum shortening of the tether, this seems to eliminate the possibility that the tether is dephosphorylated uniformly along the length. This leaves open the possibilities of losing phosphorylation from the middle outwards or from the end(s) inwards.

Titin could be a tether component as discussed above. Tether elasticity in the PEVK region is controlled by phosphorylation. PEVK phosphorylation is achieved by the action of protein kinase C, PKC (Hidalgo et al., 2009, Kotter et al., 2013). If titin is indeed a component of tethers, we might speculate that PKC is the kinase involved in phosphorylating tethers. One could test this speculation directly if one could study tethers *in vitro*. Failing that, it might be tested by adding cell-permeable inhibitors of PKC to see if they block the initial phosphorylation of tethers, tested, for example, by cutting arms in early anaphase to see if tethers are elastic, or by seeing if the inhibitor inhibits the effects of adding CalA in early anaphase.

Are tethers in cells other than crane-fly spermatocytes also dephosphorylated in late anaphase? Tethers are present in a broad range of animal cells, from cells from aquatic flatworms (*Mesostoma*) to cells from insects, spiders, marsupials, and humans, both meiotic and mitotic cells, as identified by cutting arms in anaphase and seeing that the resultant arm fragments move across the equator to their partners (Forer et al., 2017). There are no data on possible tether inelasticity in later anaphase for most of the cells, but it seems likely that they do become inelastic; otherwise, chromosomes would move backwards, away from their poles, at the end of anaphase when the poleward mitotic forces are turned off. There is evidence that tethers in PtK (marsupial) cells become inelastic as they elongate (Forer et al., 2017). However, there are no data on whether phosphorylation (or its absence) affects PtK cell tethers. These issues raise another question: Why are tethers present in mitotic/meiotic spindles? We have studied the possible role of PP1 in causing tethers to become inelastic, but why are tethers there in the first place? What do they do? There is little evidence dealing with this important question. Data from experiments on crane-fly spermatocytes suggest that tethers coordinate the movements of separating chromosomes to which they are attached.

Anaphase poleward movements of partner chromosomes are coordinated in crane-fly spermatocytes. As one example, ultraviolet light microbeam irradiation of individual kinetochore spindle fibers temporarily stops the associated chromosome from moving, but also stops the partner moving to the opposite pole. Ultraviolet microbeam irradiation of the interzone between the partners uncouples the movement: after interzonal irradiations only one chromosome stops moving, the one with the irradiated spindle fiber (Yin and Forer, 1996). While it is tempting to think the uncoupling is due to damage to tethers, there were no data presented on possible effects on tethers; i.e., there is no proof that the UV damaged tethers. More recent experiments show that when tethers are disabled, partner movements are uncoupled. These experiments dealt with a different coupling of partner chromosomes. When kinetochore microtubules are severed by laser cuts, both associated chromosomes keep moving at the same speed as prior to the laser cuts. The movements are uncoupled after tethers are severed and are uncoupled when tethers are disabled by cutting arms (Sheykhani et al., 2017; Forer et al., 2018; Forer and Berns, 2020): after severing or disabling tethers and then cutting individual kinetochore fibers, the chromosome associated with the severed microtubules accelerates while the partner moves with unchanged speed. These data show that coordination between separating partner chromosomes requires tethers, the coordination presumably arising because of the tension from the tethers on the arms of the separating chromosomes.

Experiments on *Mesostoma* spermatocytes suggest that tethers might coordinate movements between different chromosomes in the spindle. *Mesostoma* spermatocytes contain three bivalents; each has only one chiasma and two free arms. The free arms are connected by tethers, identified because arm fragments formed by cutting arms with a laser move rapidly to the partner free arm (Forer et al., 2017). After depolymerising spindle microtubules with nocodazole, each of the three bivalents stretches out between the poles and then after some minutes all three kinetochores at one pole detach from that pole and move rapidly to the other pole (Fegaras and Forer, 2018). Disabling any one of the three tethers, however, uncouples the coordination, such that after treatment with nocodazole different bivalents move to different poles, or do not move at all (Fegaras-Arch et al., 2020). Somehow tethers are necessary to coordinate the movements of these three different chromosomes.

In both these spermatocytes tethers are involved in coordination of chromosome movements, albeit with different manifestations. If tethers function in other cells as they do in these two cell types, one of their functions may involve coordinating movements of partner chromosomes or of different chromosomes. Unfortunately, we do not know more about the functions of tethers other than what was learned from these two sets of experiments.

Our overall conclusion is that when PP1 is inhibited, tethers between separating anaphase chromosomes do not become inelastic in later anaphase, as they otherwise would do. Thus, tether elasticity is controlled by phosphorylation. We suggest that tethers themselves are phosphorylated, and we suggest that tethers may contain or be composed of the elastic protein titin.

## Data Availability Statement

The raw data supporting the conclusions of this article will be made available by the authors, without undue reservation.

## Author Contributions

AF performed the experiments in the laboratory of MB and wrote drafts of the article. AA made measurements and graphed most of the cells. MB consulted on the experiments and edited drafts. All authors contributed to the article and approved the submitted version.

## Conflict of Interest

The authors declare that the research was conducted in the absence of any commercial or financial relationships that could be construed as a potential conflict of interest.
